# Assisted reproductive technology-associated risk factors for placenta accreta spectrum after vaginal delivery

**DOI:** 10.1038/s41598-024-57988-x

**Published:** 2024-03-29

**Authors:** Seung Chik Jwa, Shunsuke Tamaru, Masashi Takamura, Akira Namba, Takeshi Kajihara, Osamu Ishihara, Yoshimasa Kamei

**Affiliations:** 1 Department of Obstetrics and Gynecology, Saitama Medical University, 38 Morohongo, Moroyama, Saitama, 350-0495 Japan; 2 Kagawa Nutrition University, Saitama, Japan; 3 Department of Obstetrics and Gynecology, Jichi Medical University, Tochigi, Japan

**Keywords:** Assisted reproductive technique, Embryo transfer, Hormone replacement cycle, Placenta accreta spectrum, In vitro fertilization, Risk factors, Infertility

## Abstract

This study aimed to investigate assisted reproductive technology (ART) factors associated with placenta accreta spectrum (PAS) after vaginal delivery. This was a registry-based retrospective cohort study using the Japanese national ART registry. Cases of live singleton infants born via vaginal delivery after single embryo transfer (ET) between 2007 and 2020 were included (n = 224,043). PAS was diagnosed in 1412 cases (0.63% of deliveries), including 1360 cases (96.3%) derived from frozen-thawed ET cycles and 52 (3.7%) following fresh ET. Among fresh ET cycles, assisted hatching (AH) (adjusted odds ratio [aOR], 2.5; 95% confidence interval [CI] 1.4–4.7) and blastocyst embryo transfer (aOR, 2.2; 95% CI 1.3–3.9) were associated with a significantly increased risk of PAS. For frozen-thawed ET cycles, hormone replacement cycles (HRCs) constituted the greatest risk factor (aOR, 11.4; 95% CI 8.7–15.0), with PAS occurring in 1.4% of all vaginal deliveries following HRC (1258/91,418 deliveries) compared with only 0.11% following natural cycles (55/47,936). AH was also associated with a significantly increased risk of PAS in frozen-thawed cycles (aOR, 1.2; 95% CI 1.02–1.3). Our findings indicate the need for additional care in the management of patients undergoing vaginal delivery following ART with HRC and AH.

## Introduction

Placenta accreta spectrum (PAS) is a life-threatening complication of pregnancy that can cause massive blood loss during delivery^1–3^. This condition results from abnormal invasion of the placenta into the myometrium of the uterine wall^3^. The main risk factor for PAS is uterine scarring, e.g., as a result of previous uterine surgeries such as cesarean section and myomectomy, with the greatest risk associated with a history of multiple cesarean sections^4–6^. Also, assisted reproductive technology (ART) has been reported to be a risk factor for PAS^7,8^.

Many PAS cases are assumed to be complicated by placenta previa and are delivered via cesarean section because of uterine scarring and the requirement for prompt hysterectomy^3^. However, recent prospective population-based study demonstrated that more than half of PAS cases occurred in women without combination of placenta previa and a previous cesarean section^1^. Such cases are tended to remain undiagnosed at the time of delivery, and leading to vaginal delivery. Importantly, ART was one of risk factors for those cases^1,9^. Undiagnosed PAS is also associated with increased maternal morbidity^10^, reflecting the better management of diagnosed cases^11^.

Despite recent focus on the link between ART and lack of an antenatal PAS diagnosis, it remains unclear if the risk of PAS after ART in patients without an unscarred uterus is related to individual characteristics or to factors associated with ART, such as ovarian stimulation/endometrial preparation protocols, fertilization methods, or assisted hatching (AH). It has been reported that a non-physiological hormone environment during implantation and early pregnancy may lead to abnormal placentation^8^, suggesting that ovarian stimulation in fresh cycles and endometrial preparation protocols in frozen cycles may affect placentation and increase the risk of PAS, regardless of uterine scarring. Use of specific ovarian stimulation agents, such as clomiphene citrate (CC) and gonadotropin (Gn), has also been associated with adverse perinatal outcomes^12^ and might be associated with PAS. Similarly, a previous study reported a possible link between removing the zona pellucida (AH) and abnormal placental outcomes, including PAS^13^.

The identification of PAS risk factors is necessary to improve its early diagnosis. We therefore aimed to examine ART-related risks by carrying out a retrospective cohort study based on data from the Japanese national ART registry. We analyzed potential risk factors including the type of ovarian stimulation/endometrial preparation protocols, fertilization method, and use of AH. Furthermore, we restricted our analysis to vaginal births, to avoid any effects of uterine scarring on PAS risk.

## Results

**Figure 1:**
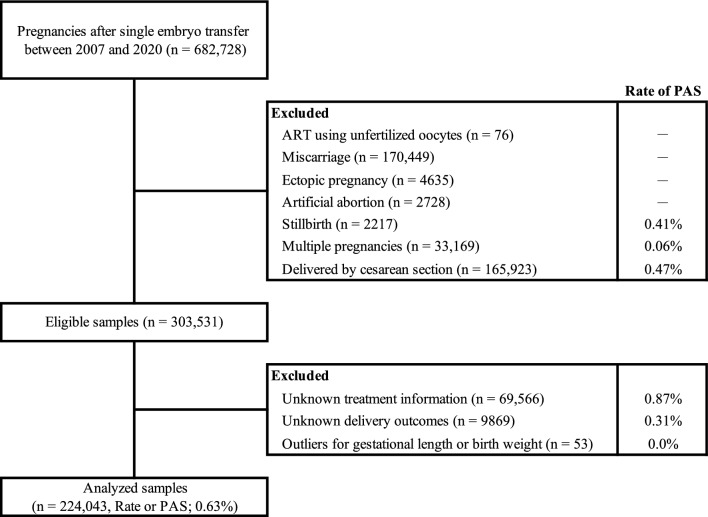
Study flow chart. PAS, placenta accreta spectrum.

A flow diagram of the sample selection process is shown in Fig. 1. There were 682,728 clinical pregnancies after single embryo transfer (ET) between 2007 and 2020. Among these, cases of ART using unfertilized oocytes (n = 76), miscarriages (n = 170,449), ectopic pregnancies (n = 4635), artificial abortions (n = 2728), stillbirth cases (n = 2217), multiple pregnancies (n = 33,169), and deliveries by cesarean section were excluded (n = 165,923). The remaining 303,531 pregnancies were considered eligible for analysis. Of these, cases with unknown treatment information or delivery outcome and outliers for gestational duration and birth weight were excluded, and 224,043 cases were finally analyzed. Among the excluded cases, the prevalence of PAS were the lowest in multiple pregnancies (0.06%) and the highest in cases with unknown treatment information (0.87%). Of cases with unknown treatment information (n = 69,566), fresh ET accounted for 4091 (5.9%), while frozen-thawed ET (FET) cycles accounted for 94.1%.

The characteristics of the sample population stratified by PAS are shown in Table 1. Overall, PAS was diagnosed in 1412 cases (0.63% of deliveries), including 1360 cases (96.3%) derived from FET cycles. The mean maternal age was 34.8 years and was comparable in PAS and non-PAS patients. The incidence of PAS was significantly higher in patients with tubal or endometriosis or male factor or unexplained infertility compared with patients without either condition. In addition, there was a significant increasing trend in PAS incidence with successive study years (*P* for trend < 0.001).

**Table 1:** Characteristics of the sample population stratified by placenta accreta spectrum (n = 224,043).

Variables	Placenta accreta (−)	Placenta accreta (+)	*P* value^a^
	n = 222,631	n = 1412	
Mean age (years)	34.8 (3.9)	34.8 (4.0)	0.47
< 30	20,813 (99.4)	134 (0.64)	0.99
30–34	81,189 (99.4)	519 (0.64)	
35–39	94,260 (99.4)	593 (0.63)	
≥ 40	26,369 (99.4)	166 (0.63)	
Tubal factor infertility			
Yes	32,837 (99.2)	257 (0.78)	< 0.001
No	189,794 (99.4)	1155 (0.60)	
Endometriosis			
Yes	14,839 (99.2)	120 (0.80)	0.006
No	207,792 (99.4)	1292 (0.62)	
Antisperm antibody			
Yes	1229 (99.1)	11 (0.89)	0.25
No	221,402 (99.4)	1401 (0.63)	
Male factor			
Yes	62,069 (99.3)	459 (0.73)	< 0.001
No	160,562 (99.4)	953 (0.59)	
PCOS			
Yes	8804 (99.4)	56 (0.63)	0.98
No	213,827 (99.4)	1356 (0.63)	
Others			
Yes	98,993 (99.5)	491 (0.49)	< 0.001
No	123,638 (99.3)	921 (0.74)	
Unexplained			
Yes	31,555 (99.0)	319 (1.0)	< 0.001
No	191,076 (99.4)	1093 (0.57)	
Fresh ET	58,380 (99.9)	52 (0.09)	< 0.001
Frozen-thawed ET	164,251 (99.2)	1360 (0.82)	
Year			
2007	3554 (99.97)	1 (0.03)	< 0.001^b^
2008	5382 (99.9)	7 (0.13)	
2009	6993 (99.86)	10 (0.14)	
2010	7374 (99.97)	2 (0.03)	
2011	8471 (99.8)	16 (0.19)	
2012	12,490 (99.8)	20 (0.16)	
2013	15,012 (99.6)	55 (0.37)	
2014	18,003 (99.5)	87 (0.48)	
2015	20,493 (99.6)	80 (0.39)	
2016	22,641(99.3)	153 (0.67)	
2017	24,081 (99.2)	188 (0.77)	
2018	25,191 (99.1)	231 (0.91	
2019	26,459 (98.9)	283 (1.1)	
2020	26,487 (99.0)	179 (1.0)	

Data presented as mean (standard deviation) for continuous variables and n (%) for dichotomous variables; percentages presented in rows for comparison.

*ET* Embryo transfer, *PCOS* Polycystic ovary syndrome.

^a^*P* values were calculated using the Student’s *t* test, chi-squared test or Fisher’s exact test.

^b^*P* value for trend assessed with linear regression.

PAS incidence and the associated odds ratios (ORs) for ART treatment factors among patients undergoing fresh cycles are shown in Table 2. PAS was reported in 52 of 58,435 deliveries (0.09%). Among these cases, AH (0.21% [17/7986] vs. 0.07% [28/40971], adjusted OR = 2.5; 95% confidence interval [CI] 1.4–4.7) and blastocyst ET (0.15% [26/17215] vs. 0.06% [26/41033], adjusted OR = 2.2; 95% CI 1.3–3.9) were associated with significantly increased risks of PAS.

**Table 2:** Assisted reproductive technology treatment factors and odds ratios for placenta accreta spectrum in patients undergoing fresh cycles (n = 58,432).

Variables	Placenta accreta (−)	Placenta accreta (+)	*P* value^a^	Crude OR (95% CI)	Adjusted OR (95% CI)^b^
	n = 58,380	n = 52			
Ovarian stimulation protocols					
Natural	6561 (99.95)	3 (0.05)	0.008	Reference	Reference
CC alone	6259 (99.98)	1 (0.02)		0.35 (0.04 to 3.4)	0.37 (0.04 to 3.6)
CC + Gn	7588 (99.99)	1 (0.01)		0.29 (0.03 to 2.8)	0.30 (0.03 to 2.9)
Gn	1071 (99.81)	2 (0.19)		4.1 (0.68 to 24.5)	3.9 (0.65 to 23.7)
GnRH agonist	19,768 (99.9)	24 (0.12)		2.7 (0.80 to 8.8)	2.6 (0.77 to 8.7)
GnRH antagonist	10,985 (99.9)	16 (0.15)		3.2 (0.93 to 10.9)	3.0 (0.86 to 10.4)
Others	6148 (99.92)	5 (0.08)		1.8 (0.42 to 7.4)	1.6 (0.38 to 6.7)
Number of oocytes retrieved					
< 4	25,212 (99.9)	16 (0.06)	0.33	Reference	Reference
4–9	22,278 (99.9)	24 (0.11)		1.7 (0.90 to 3.2)	1.7 (0.87 to 3.2)
10–15	8606 (99.9)	10 (0.12)		1.8 (0.83 to 4.0)	2.0 (0.90 to 4.5)
> 15	2284 (99.9)	2 (0.09)		1.4 (0.32 to 6.0)	1.7 (0.39 to 7.7)
Fertilization methods					
IVF	27,096 (99.9)	24 (0.09)	0.91	Reference	Reference
ICSI	24,242 (99.9)	23 (0.09)		1.1 (0.60 to 1.9)	1.1 (0.57 to 2.0)
Split ICSI	6713 (99.9)	5 (0.07)		0.84 (0.32 to 2.2)	0.86 (0.33 to 2.3)
Others	329 (100)	0 (0)		–	–
Embryo stage at transfer					
Early cleavage	41,007 (99.9)	26 (0.06)	0.18	Reference	Reference
Blastocyst	17,189 (99.9)	26 (0.15)		**2.4 (1.4 to 4.1)**	**2.2 (1.3 to 3.9)**
Others	184 (100)	0 (0)		–	–
	(n = 48,912)	(n = 45)			
Assisted hatching (−)^c^	40,943 (99.9)	28 (0.07)	< 0.001	Reference	Reference
Assisted hatching (+)^c^	7969 (99.8)	17 (0.21)		**3.1 (1.7 to 5.7)**	**2.5 (1.4 to 4.7)**

Patient numbers represented as n (%); significantly increased or reduced odds indicated by bold type.

*CC* Clomiphene citrate, *CI* Confidence interval, *Gn* Gonadotropin, *GnRH* Gonadotropin-releasing hormone, *IVF* In vitro fertilization, *ICSI* Intracytoplasmic sperm injection, *OR* Odds ratio.

^a^*P* values calculated using the chi-squared test or Fisher’s exact test.

^b^Adjusted for maternal age, infertility diagnosis, and year. Maternal age categorized into 5-year age groups.

^c^Samples between 2010 and 2020.

For frozen cycles, the prevalence of PAS varied significantly depending on the endometrial preparation protocol used, and use of AH (Table 3). FET with hormone replacement cycles (HRCs) was associated with the highest incidence of PAS, which occurred in 1.4% of all vaginal deliveries following FET with HRC (1258/91,418) compared with 0.11% of vaginal deliveries after FET with natural cycles (55/47,936) (*P* value < 0.001). The risk of PAS was thus significantly higher after FET with HRC than after natural-cycle FET (adjusted OR = 11.4; 95% CI 8.7–15.0). Further, albeit lower compared with HRC, AH (adjusted OR = 1.2; 95% CI 1.02–1.3) also increased the risks of PAS.

**Table 3:** ART Treatment factors and odds ratios for placenta accreta spectrum in patients undergoing frozen-thawed cycles (n = 165,611).

Variables	Placenta accreta (–)	Placenta accreta (+)	*P* value^a^	Crude OR (95% CI)	Adjusted OR (95% CI)^b^
	n = 164,251	n = 1360			
Embryo stage at transfer					
Early cleavage	19,997 (99.1)	180 (0.89)	0.1	Reference	Reference
Blastocyst	143,815 (99.2)	1173 (0.81)		0.91 (0.77 to 1.1)	0.89 (0.76 to 1.04)
Others	439 (98.4)	7 (1.6)		1.8 (0.83 to 3.8)	1.7 (0.79 to 3.6)
Endometrial preparation protocols^c^	(n = 150,566)	(n = 1337)			
Natural	47,881 (99.9)	55 (0.11)	< 0.001	Reference	Reference
CC alone	913 (99.8)	2 (0.22)		1.9 (0.46 to 7.8)	2.0 (0.48 to 8.2)
CC + Gn	1053 (99.7)	3 (0.28)		2.5 (0.77 to 7.9)	2.4 (0.76 to 7.8)
Gn	1177 (99.9)	1 (0.08)		0.74 (0.10 to 5.3)	0.73 (0.10 to 5.3)
HRC	90,160 (98.6)	1258 (1.4)		**12.1 (9.3 to 15.9)**	**11.4 (8.7 to 15.0)**
Others	9382 (99.8)	18 (0.19)		1.7 (0.98 to 2.8)	**1.7 (1.01 to 2.9)**
	(n = 157,790)	(n = 1349)			
Assisted hatching (−)^d^	51,119 (99.3)	367 (0.71)	< 0.001	Reference	Reference
Assisted hatching (+)^d^	106,671 (99.1)	982 (0.91)		**1.3 (1.1 to 1.4)**	**1.2 (1.02 to 1.3)**

Patient numbers represented as n (%); significantly increased or reduced odds indicated by bold type.

*CC* Clomiphene citrate, *CI* Confidence interval, *Gn* Gonadotropin, *HRC* Hormone replacement cycle, *OR* Odds ratio.

^a^*P* values calculated using the chi-squared test or Fisher’s exact test.

^b^Adjusted for maternal age, infertility diagnosis, and year. Maternal age was categorized into 5-year age groups.

^c^Samples between 2012 and 2020.

^d^Samples between 2010 and 2020.

We conducted subgroup analyses evaluating whether the association between AH and PAS differed by different implantation situations (Table 4). Interestingly, significant associations between AH and PAS were only observed among cleavage stage ET cycles, while no significant increased risks of AH were observed both among fresh and frozen cycles in frozen cycles. Adjusted OR of AH were higher in fresh cleavage ET cycles (adjusted OR = 3.3; 95% CI 1.4–8.0) compared with FET cycles (adjusted OR = 2.3; 95% CI 1.6–3.2), but there was no significant interaction between AH and fresh/frozen status (*P* for interaction = 0.21).

**Table 4:** Subgroup analysis: adjusted odds ratios of assisted hatching for placenta accreta spectrum stratified by fresh and frozen cycles.

	Fresh cycles	Frozen cycles	P for interaction^b^
	Adjusted OR (95% CI)^a^	Adjusted OR (95% CI)^a^	
Cleavage stage embryo transfer			
Assisted hatching (−)	Reference	Reference	0.21
Assisted hatching (+)	**3.3 (1.4 to 8.0)**	**2.3 (1.6 to 3.2)**	
Blastocyst stage embryo transfer			
Assisted hatching (−)	Reference	Reference	0.43
Assisted hatching (+)	1.4 (0.59 to 3.5)	1.04 (0.91 to 1.2)	

Significantly increased or reduced odds are indicated in bold type.

*CI* Confidence interval, *OR* Odds ratio, *PAS* Placenta accreta spectrum.

^a^Adjusted for maternal age, infertility diagnosis, and year. Maternal age was categorized into 5-year age groups.

^b^*P* value for interaction between assisted hatching and frech/frozen status in adjusted model including maternal age, infertility diagnosis and year. Maternal age was categorized into 5-year age groups.

## Discussion

### Main findings

Our results showed that most cases of PAS (96.3%) occurred in patients who had undergone FET. Furthermore, among the different endometrial preparation protocols used in FET cycles, HRC was associated with the highest incidence of PAS. Both HRC and AH (to a lesser extent) were associated with significantly increased risks of PAS following FET cycles, while AH and blastocyst ET were significantly associated with increased the risks of PAS in patients undergoing fresh ET cycles. Subgroup analysis demonstrated the significant associations of AH was only observed in cleavage stage ET without interaction between fresh/frozen cycles.

### Strengths and limitations

The main strength of this study was the large number of samples analyzed, which allowed us to conduct a meaningful investigation of the factors affecting this relatively rare condition. All ART cycles carried out in Japan are registered in the Japanese ART registry, with high compliance, and registration up to and including delivery outcomes is mandatory. The inclusion of detailed treatment information in the registry thus allowed us to explore the associations between individual ART treatment factors and the risk of PAS.

In terms of limitations, the registry lacks important information relating to the risk of PAS, such as any history of previous uterine surgery, including cesarean section; however, we assume that most of those cases would have undergone cesarean section. By restricting the analysis to vaginal deliveries, which were less likely to have a history of cesarean section, we believe that we were able to remove the effect of this important confounder. Notably however, this study may have been affected by selection bias. The rate of PAS differed significantly in relation to the exclusion factors, and the rate of PAS for the initial source population excluding miscarriage, ectopic pregnancy, and artificial abortion (allowed only before 22 weeks of gestation in Japan) was lower (0.55%) than the analyzed sample (0.63%). The exclusion of cases with unknown treatment information in particular might have introduced bias for the analyzed results. The registry also lacks information on parity, smoking status, and previous miscarriages, and residual confounding for the observed associations may thus have remained. Furthermore, the indications for fresh/frozen ETs were not recorded in the registry, and individual characteristics such as previous treatments and adverse previous pregnancy outcomes might have affected the choice of treatment. In addition, most PAS diagnoses relied on a clinical diagnosis by obstetricians (unless the patient underwent hysterectomy), leading to a possible bias in the results; however, although definitions of PAS may vary among studies^9,14^, an excessively adherent placenta may result in clinical problems, regardless of later histological confirmation of PAS status, and such definitions may thus only be important insofar as they reflect a clinical entity, as noted previously^15^. Finally, although we found significant associations between specific ART treatments and PAS, the reported incidence of PAS was very low (0.63%), and the absolute risk difference was very low, indicating the need for caution when interpreting the results.

### Interpretation

Although previous studies have identified ART as a risk factor for PAS, the detailed ART-related treatment factors and their associations with PAS have rarely been investigated. Among these factors however, FET has been reported as a risk factor for PAS. Ishihara et al. reported that FET increased the risk of PAS among singleton live birth pregnancies following single ET, compared with fresh ET (0.26–0.27% vs. 0.08–0.09%)^7^, while Kaser et al. conducted a case–control study and identified FET as a strong independent risk factor for PAS in patients undergoing ART (adjusted OR, 3.20, 95% CI, 1.14–9.02)^8^. Although these studies did not exclude cesarean sections and it was unclear whether the risk was related to the process of freeze/thawing or to the method of endometrial preparation, these findings were largely consistent with the current findings. Previous reports showed greater uterine perfusion in pregnancies after FET compared with fresh ET; the uterine artery Doppler pulsatility index was significantly lower in pregnancies after FET than after fresh ET, especially from the early first to the late third trimester, and these differences were associated with infant birth weight^16,17^. The difference in placental perfusion in pregnancies after FET compared with fresh ET might be associated with abnormal placentation and the risk of PAS.

Our findings demonstrated an association between PAS and endometrial preparation methods, especially HRC, in line with previous studies. Sakai et al. also identified an association between PAS and HRC in a study of 87 patients undergoing ET^18^, indicating that endometrial preparation by HRC may contribute to the risk of PAS by affecting the endometrial environment.

Progesterone and estradiol play critical roles in placental development, and previous studies reported that non-physiological hormone levels during HRC had adverse effects on placental development^18,19^. Progesterone induces decidualization of the endometrial stromal cells and regulates extravillous trophoblast (EVT) invasion, which enables the developing placenta and fetus to access the maternal blood supply. Abnormal progesterone levels in early pregnancy can thus lead to invasion defects of the EVT^20^, while decreased progesterone levels in early pregnancy can lead to PAS as a result of the failure of normal decidualization and excessive EVT invasion^21,22^. Furthermore, low serum levels of estradiol and a thin endometrium, as typically found in HRC, have also been associated with PAS risk^8^.

In addition, in contrast with natural cycles, HRC does not create a corpus luteum (CL). Importantly, in addition to producing estrogen and progesterone, the CL also releases vasoactive products such as relaxin^23^, and several studies have demonstrated that relaxin induces the production of the decidualization markers insulin-like growth factor-binding protein 1 and prolactin^24,25^. These observations suggest that the decidualization process may be attenuated in HRC by the lack of a CL, potentially leading to PAS.

Our results implicated AH as a risk factor for PAS following both fresh and frozen ET cycles especially in early cleavage stage ETs. Notably however, AH was not identified as a risk factor for PAS in a previous case–control study^8^, while a previous retrospective cohort study also showed that adverse placental outcomes (including but not restricted to PAS) were not linked to AH^13^, and a recent study comparing placental histology in a historic cohort that had undergone fresh cycles with or without AH showed no significant difference in the incidence of PAS^26^. Although these earlier studies included smaller sample sizes than the current study, further investigations are required to clarify this relationship.

Considering the increased number of FETs and subsequent neonates born after ART, the prevalence of PAS might be expected to increase as our study demonstrated. Our results suggest that specific ART techniques may increase the risk of PAS, even among vaginal deliveries. Based on a recent reports suggesting an association between PAS and amniotic fluid embolism, the increased use of ART techniques might also be associated with this fatal maternal complication, highlighting the need to closely monitor patients with PAS following ART^2,27^.

### Conclusion

In conclusion, PAS occurred in 1.4% of singleton vaginal deliveries following ART employing FET with HRC. HRC was the greatest risk factor for PAS, while AH was also associated with an increased risk of PAS following both fresh and frozen cycles. Further studies are warranted to investigate the effects of the hormonal environment during implantation and the potential role of AH in relation to abnormal placentation.

## Methods

This was a registry-based retrospective cohort study using data from the Japanese national ART registry, managed by the Japan Society for Obstetrics & Gynecology. The registry was established as a mandatory online reporting system in 2007. Japanese ART facilities are required to register all treatment cycles, and patients are unable to receive government subsidies if their ART facility does not complete cycle registration. The registry consists of cycle-specific information, including patient characteristics such as patient age and infertility diagnosis. Additional information includes treatment details such as fertilization method (namely in vitro fertilization [IVF] or intracytoplasmic sperm injection [ICSI]), embryo stage at transfer, number of embryos transferred, and pregnancy/delivery outcomes (including complications). The use of donated oocytes is prohibited in Japan, and all cycles are therefore autologous. Preimplantation genetic testing for chromosomal aneuploidy was not practiced in Japan during the study period. This study was approved by the institutional review board at Saitama Medical University (Approval number: 2021–016, September 2021) and the ethics committee of the Japan Society for Obstetrics & Gynecology (Approval number: 2021–11, March 2022). The need for informed consent was waived by the institutional review board of Saitama Medical University because the study used anonymized data. The study was conducted in accordance with Japanese law and the STROBE guidelines. All methods were performed in accordance with the relevant guidelines and regulations.

To avoid any effect of uterine scarring on the risk of PAS, we specifically restricted samples to vaginal delivery cases. Singleton live births delivered after 22 weeks of gestation via vaginal delivery following single ET were included in the study.

The main outcome was the occurrence of PAS, including placenta percreta and increta. The identification of PAS in cases without hysterectomy depended on its clinical diagnosis by obstetricians (including manual removal of the placenta impossible or incomplete after delivery, despite active management in third-stage labor/ massive bleeding of the site of placental insertion after difficult manual delivery of placenta)^14^. Cases of retained placenta were excluded from the outcome. Cases with hysterectomy were based on pathological diagnosis.

In addition to the above information, detailed treatment information was available from the registry, including ovarian stimulation/endometrial preparation protocols. Details of embryo stage at transfer (early cleavage or blastocyst) and the use of AH were also obtained. Registry information on AH was available from 2010, and HRC for endometrial preparation was available from 2012.

For the analyses, we first compared baseline characteristics and treatment information between cases with and without PAS using Student’s *t* tests for continuous variables and χ^2^ or Fisher’s exact test for categorical variables as appropriate. We also investigated the treatment factors associated with PAS using bivariate and multiple logistic regression analyses (separate analyses for fresh and frozen cycles) and calculated the ORs and 95% CIs for PAS risk For fresh cycles, the analyzed treatment factors included ovarian stimulation protocol (e.g., natural, mild ovarian stimulation using CC alone, CC + Gn, Gn alone, and Gn-releasing hormone agonist and antagonist protocols), numbers of oocytes retrieved (categorized as < 4, 4–9, 10–15, and > 15) as a proxy for elevated estradiol levels during implantation and early pregnancy, fertilization method (IVF, ICSI, split ICSI, and others), embryo stage at transfer (early cleavage, blastocyst, and others), and AH. For frozen cycles, we investigated endometrial preparation protocols (natural, HRC, ovarian stimulation using CC, CC + Gn, and Gn), embryo stage at transfer, and AH. Each treatment factor was adjusted for confounders including maternal age (categorized into 5-year age groups), infertility diagnosis, and the year of registered cycles. Because information on HRC was only available from 2012, the risks related to endometrial preparation protocols were calculated in the subgroup of cases between 2012 and 2020. Similarly, information for AH was only available from 2010, and the risks of AH in fresh and FET cycles were therefore calculated in the subgroup of cases between 2010 and 2020. Then, to evaluate observed significant associations between AH and PAS, we conducted additional subgroup analyses to determine if the observed associations varied between specific subgroups, namely between fresh and FET cycles, and between early cleavage and blastocyst ETs. Statistical interactions between AH and fresh/frozen status for PAS were evaluated. All analyses were conducted using Stata MP, version 17.0 (StataCorp LLC, College Station, TX, USA). A two-tailed *P* value < 0.05 was considered statistically significant.

## Data Availability

The datasets analyzed during the current study are not publicly available because they include specific care-required personal information, but are available from the corresponding author on reasonable request.
